# The three regimes of spatial recovery

**DOI:** 10.1002/ecy.2586

**Published:** 2019-02-01

**Authors:** Yuval R. Zelnik, Jean‐François Arnoldi, Michel Loreau

**Affiliations:** ^1^ Centre for Biodiversity Theory and Modelling Theoretical and Experimental Ecology Station CNRS and Paul Sabatier University 09200 Moulis France; ^2^ Trinity College Dublin School of Natural Sciences Zoology Building Dublin 2 Ireland

**Keywords:** dispersal, ecological stability, habitat fragmentation, local extinctions, metacommunity, metapopulation, pulse disturbance

## Abstract

An enduring challenge for ecology is identifying the drivers of ecosystem and population stability. In a spatially explicit context, key features to consider are landscape spatial structure, local interactions, and dispersal. Substantial work has been done on each of these features as a driver of stability, but little is known on the interplay between them. Missing has been a more integrative approach, able to map and identify different dynamical regimes, predicting a system's response to perturbations. Here we first consider a simple scenario, i.e., the recovery of a homogeneous metapopulation from a single localized pulse disturbance. The analysis of this scenario reveals three fundamental recovery regimes: *Isolated Regime* when dispersal is not significant, *Rescue Regime* when dispersal mediates recovery, and *Mixing Regime* when perturbations spread throughout the system. Despite its simplicity, our approach leads to remarkably general predictions. These include the qualitatively different outcomes of various scenarios of habitat fragmentation, the surprising benefits of local extinctions on population persistence at the transition between regimes, and the productivity shifts of metacommunities in a changing environment. This study thus provides context to known results and insight into future directions of research.

## Introduction

How can dispersal, ecosystem size, and local dynamics interact to determine recovery from a disturbance? This question is fundamental to ecology, not only due to its relevance for conservation and management, but because it connects key concepts of ecology, such as stability, landscape, metapopulations, and disturbance.

Dispersal plays a fundamental role in all aspects of ecology, affecting the stability of populations (Abbott 2011), biodiversity patterns (Haegeman and Loreau 2014), trophic interactions (McCann et al. 2005) and evolutionary dynamics (Baskett et al. 2007). Dispersal is often studied because of two main effects it has on ecosystems: sustaining diversity (Kerr et al. 2002) and generating population synchrony (Lande et al. 1999, Abbott 2011). When dispersal is weak, it can promote diversity, allowing populations to benefit from spatial insurance effects, whereby good patches prevent local extinctions in less favored locations (Loreau et al. 2003). This effect is fundamental in the context of biodiversity loss caused by human‐induced landscape fragmentation, which impedes dispersal (Burkey 1989, Fischer and Lindenmayer 2007). Dispersal, however, is not always beneficial. Strong dispersal may synchronize population dynamics and cause global extinctions. It can inhibit spatial insurance effects, causing generalist species to competitively exclude specialists (Abbott 2011). The opposite scenarios described, i.e., extinctions caused by dispersal limitations, or global synchrony due to strong dispersal, are extreme cases where there is a clear separation of timescales between the local dynamics and the time it takes to disperse across the system. In between is an intermediate regime without a clear separation of timescales, which has not been investigated much or even well defined.

Not all relevant spatial aspects of ecosystems are centered on dispersal and interactions across space. Sheer size is also important as spatial processes are effectively mediated by the system size (Galiana et al. 2018). Larger regions can allow for substantial spatial heterogeneity, from asynchrony due to nonlinear local dynamics or disturbance regimes (Bjørnstad et al. 1999, Gouhier and Guichard 2007), to an imposed structure due to topography or climatic gradients (Qian et al. 2009). Spatial averaging over such heterogeneities has led to many well‐known concepts in ecology, such as the species–area relationship (Connor and McCoy 1979) and landscape equilibrium (Turner 1989).

The ecological concepts discussed above, such as synchrony and spatial averaging, are non‐trivial due to local dynamics that act in conjunction with spatial effects. Ecology has long focused on local non‐spatial behavior, with central issues such as the diversity–stability debate (MacArthur 1955, May 1973, McCann 2000, Loreau and de Mazancourt 2013) largely addressed by focusing on local interactions between species. Assumptions on local dynamics vary greatly from linear behavior around an equilibrium to highly nonlinear dynamics far from it. This is evident in stability research where noisy time series are typically assumed to be close to equilibrium (Ives 1999), while catastrophic regime shifts are inherently nonlinear (Scheffer and Carpenter 2003). When considering spatial aspects, however, most studies implicitly or explicitly assume linear behavior, while research into nonlinear behavior is mostly focused on specific scenarios such as emergent stationary spatial patterns in drylands (von Hardenberg et al. 2010) or chaotic behavior of algae blooms (Franks 1997).

Stability is a central notion in ecology, and it can be defined in various ways, which are typically context dependent (Grimm and Wissel 1997). Nevertheless, the concept of stability comes down fundamentally to the ability of the system to recover from a perturbation (Arnoldi et al. 2018), which may be affected by the timing of the perturbation (e.g., constant or single event), the dynamical aspect considered (e.g., rate of convergence or disturbance strength withstood), or the central measure recovered (e.g., biodiversity or overall biomass).

Recent work has investigated the stability of populations and ecosystems in a spatial context, by explicitly considering the issues of dispersal, system size and local dynamics (Yaari et al. 2012, Dai et al. 2013, Plitzko and Drossel 2015, Fox et al. 2017, Gilarranz et al. 2017, Wang et al. 2017). The scenarios considered, however, are often quite specific, and it is difficult to draw general conclusions from them. Moreover, since there is no clear framework in which to understand the phenomena described, results are hard to compare, limiting the potential for synthesis. Here we propose that answering the preliminary question of how dispersal, ecosystem size, and local dynamics interact to determine recovery from a disturbance provides a unifying framework to understand and compare the dynamical behavior of spatially extended ecosystems. We first address this preliminary question in a simplified setting, i.e., a metapopulation subject to a disturbance (a sudden change in abundance) in a uniform one‐dimensional landscape. We monitor the time needed for the system to return to its pre‐disturbed state. This notion is easily measured and understood, while having clear relations to other notions of stability (Arnoldi et al. 2016). This allows us to draw an exhaustive map of dynamical behavior, predicting the transitions between three qualitatively different recovery regimes: Isolated Regime (IR), Rescue Regime (RR), and Mixing Regime (MR). In IR, dispersal is not essential for recovery. In RR, propagation of biomass is key for recovery. Finally, in MR, recovery occurs after the disturbance's effect has spread throughout the system.

We then translate this approach to more complex ecological settings: early warning signals of catastrophic transitions, the interplay between local extinctions and metapopulation persistence, and productivity of metacommunities in a changing environment. Our approach thus defines a powerful methodology for the analysis of spatial ecosystems and proposes new directions of research for fundamental and applied ecology.

## Conceptual Framework

We look for the general mechanisms that underlie spatial dynamics of ecosystems. In this section, we reveal them in the simplest possible setting, focusing on the behavior of a single species that is at a stable equilibrium before a disturbance occurs. As we shall see in *Examples*, the basic recovery regimes at play in this specific setting also play out in systems with more complex spatial structure, multiple interacting species, and even in systems that do not have a stable equilibrium state. We use a partial differential equation as a mathematical description of our system, so that its behavior is governed by the combination of local interactions of individuals, and dispersal in space of these individuals. The dynamics of the system read

(1) $$\partial_{T}N=rNF\left(N\right)+d\nabla^{2}N$$

where N is the density of individuals at a location, r the characteristic, local, dynamical rate of growth, and d the dispersal coefficients. The expression $\partial_{T}$ denotes the time derivative, $\nabla^{2}$ the diffusion operator (in a one‐dimensional system, it amounts to the second spatial derivative $\partial_{\mathrm{XX}}$), and F describes the local (non‐dimensional) growth rate of the population, which we assume has an equilibrium solution for N=K. We denote the size of the system by L.

**Figure 1:**
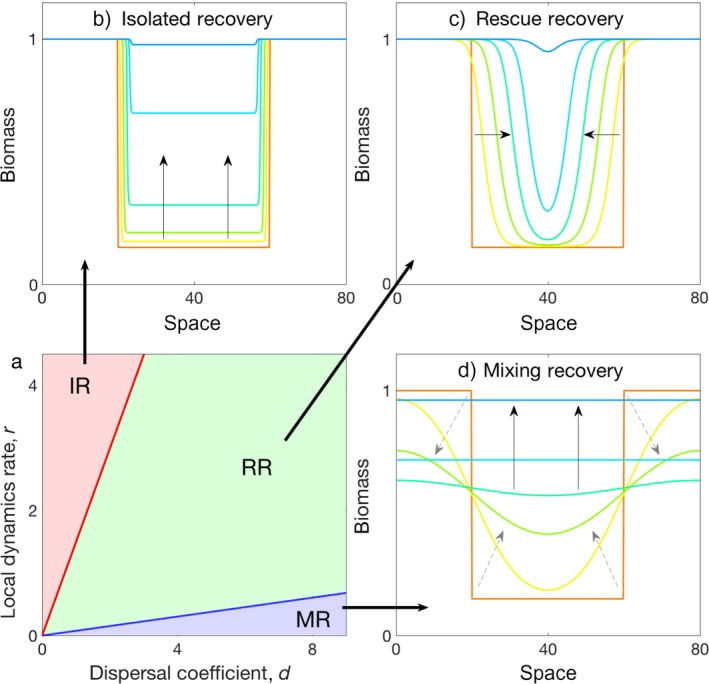
Three recovery regimes across the space of possible systems. (a) Parameter space of local dynamics rate r vs. dispersal coefficient d, noting the three regimes of recovery: isolated (IR), rescue (RR), and mixing (MR). (b–d) Spatial profiles of biomass at different times (shown by different colors), demonstrating the recovery under each regime, with black arrows showing the direction of recovery. The system's equilibrium of N=1 is disturbed by setting one‐half the system to N=0.15 (orange), with different colors (yellow to blue) showing the recovery over time. (b) IR (d=0.01,r=4) shows recovery due to local processes alone. (c) In RR (d=1,r=1), recovery due to spatial spread dominates. (d) In MR (d=8,r=0.01), the system first homogenizes (gray dashed arrows) and then, at a later time, the recovery takes place.

An important feature of the system is the nonlinearity of its local dynamics, in particular the difference in rates between the dynamics close and far from equilibrium. To explore this feature, we focus on one specific type of local growth rate function $F\left(N\right)=\left(1-N/K\right){\left(N/K\right)}^{\gamma }$, where a large γ leads to higher nonlinearity and slower dynamics when far from the equilibrium N=K. For γ=0, this is logistic growth in space, a well‐studied model (Lewis and Kareiva 1993, Gandhi et al. 2016) within the general framework of Fisher fronts, representing the invasion dynamics of a stable state into a metastable one (van Saarloos 2003). See also Fig. 1c for an example of a front. For large values of γ, the dynamics resemble more that of bistable dynamics in space (Lewis and Kareiva 1993, Bel et al. 2012) where a stable state can invade another stable state. We choose this functional form for the growth rate as it allows us to more clearly explore the role of different mechanisms, under two main assumptions: (1) the system always recovers from a single disturbance and (2) local dynamics around the equilibrium are faster than those far from equilibrium, thus emphasizing the detrimental effects of locally strong disturbances. We further comment on these assumptions in the discussion, and in Appendix S4.

Having defined the system, we now study its recovery from a single, localized, pulse disturbance. In Fig. 1 we show the different possible responses of the system, and how these map out in the parameter space spanned by r and d. We see that the recovery follows one of three scenarios: Isolated Regime (IR), Rescue Regime (RR), and Mixing Regime (MR).

For weak dispersal d and fast local dynamics r, the system is in IR: the recovery of each location occurs without any relation to other regions. We quantify this notion by the ratio between the biomass recovery when dispersal d=0 and when d>0, within the same time frame. On the other hand, for large d and low r values, the system is in MR: the recovery of all the locations of the system effectively occurs together; dispersal homogenizes the system before local recovery processes take place. This can be quantified by defining a mixing time, when all locations of the system reach similar biomass densities (within some threshold), and attributing all biomass recovered after this time to MR. Finally, for intermediate values of d and r, the system is in RR, where undisturbed regions aid the disturbed ones via spatial spread processes. This occurs when non‐spatial dynamics of the disturbed region are sufficiently slow, so that spatial recovery processes (e.g., front propagation) can come into play, yet dispersal is not strong enough to homogenize the system. While it is possible to define this recovery directly, it is more simply defined as any recovery that is not attributed to IR or MR.

**Figure 2:**
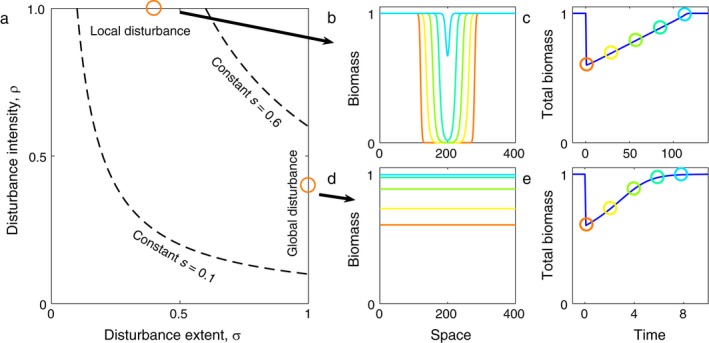
Space of possible disturbances, and the recovery that follows them. (a) Parameter space of disturbances: spatial extent (σ) vs. local intensity (ρ). The disturbance's overall strength is s=σρ, where dashed curves show constant values of s. (b, c) Recovery following a local disturbance (σ=0.4,ρ=1). (d, e) Recovery following a global disturbance (σ=1,ρ=0.4). Spatial profiles of biomass at different times (orange through blue) are shown in panels b and d, while, in panels c and e, the overall biomass over time is shown (with circles of corresponding colors to the profiles shown in panels b and d).

An important determinant of recovery is the disturbance itself. Here there are essentially three disturbance properties to consider: overall strength s (total biomass removed), spatial extent σ, and intensity ρ=s/σ. As shown in Fig. 2, these properties define a parameter space in which we can map all possible disturbances, and the system response they induce. For a given disturbance strength s there are two extreme cases (orange circles in Fig. 2a): global disturbance (maximal extent σ), and localized disturbance (maximal intensity ρ). These contrasting cases are met with very different recovery behaviors. The recovery timescale is much shorter in response to a global disturbance than to a localized one, and the transient spatial profiles are strikingly different: in Fig. 2b we see the formation of fronts, while Fig. 2d only shows a uniform increase of biomass in time.

## Recovery Regimes

**Figure 3:**
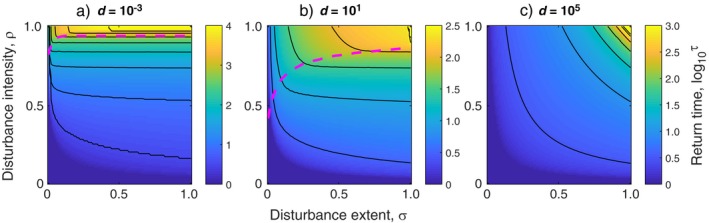
Return time across the disturbance space for three levels of dispersal. Each panel shows return time $\log_{10}\tau$ as a function of disturbance extent σ and intensity ρ. Dispersal increases from left to right as $d=\{10^{-3},10^{1},10^{5}\}$. (a, b) IR and RR below and above magenta line (low and high ρ), respectively; (c) MR. Black lines show contours at 0.5 intervals. Return time defined as the duration needed for the system to recover to 99% of its equilibrium biomass.

To complement the qualitative picture of the previous section we now give a quantitative description of the recovery process. To quantify recovery, we study return time, defined as the duration needed for the system to recover to 99% of its equilibrium biomass. In Fig. 3, we map out return time as a function of disturbance extent, disturbance intensity, and dispersal. While return time grows with all three disturbance properties, ρ, σ, and s, there are substantial differences in how they affect return time as we increase dispersal.

When dispersal is weak (Fig. 3a), the dynamics are mostly governed by local processes. If the intensity ρ is not too large, then local recovery dynamics are fast, so that one can ignore spatial effects, and the return time is effectively controlled by ρ. However, for high ρ, there is a switch in behavior, and the spatial extent σ becomes dominant in determining return time. This is because local recovery is now slow enough to allow for spread processes to become relevant, and their timescale is largely set by σ. For intermediate dispersal (Fig. 3b), the spatial processes can have more of an effect, so that σ becomes important at lower values of ρ. Hence, we see here the same picture as in Fig. 3a, except that the switch in behavior occurs for lower ρ.

Finally, for strong dispersal (Fig. 3c) spatial processes are fast enough to homogenize the system before local dynamics become significant. Therefore the spatial structure of the disturbance is irrelevant, only its overall strength s is important.

We can understand the effect of disturbance properties by knowledge of the recovery regimes (magenta lines in Fig. 3 outline regime boundaries). In IR (low ρ, low and intermediate d), the recovery is entirely local so that return time is controlled by ρ. In RR (high ρ, low and intermediate d), spatial spread processes are responsible for recovery so that return time is controlled by σ. Finally, in MR (high d), recovery only takes place after the disturbance has spread throughout the system, hence s controls return time.

**Figure 4:**
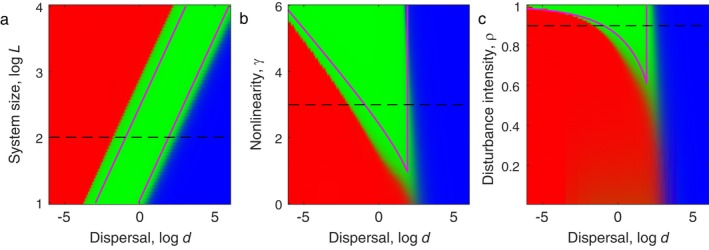
Effects of dispersal vs. system size, nonlinearity of local dynamics and disturbance intensity on the three recovery regimes. Different regimes, IR (red), RR (green), and MR (blue), shown on three parameter spaces. The parameter spaces span the dispersal coefficient d on the *x*‐axis (in log scale), and the (a) system size L, (b) nonlinearity of local dynamics γ, and (c) intensity of disturbance ρ, on the *y*‐axis. Magenta lines show the prediction for the transitions between regimes (defined by Eq. (2), see Recovery regimes for details). Dashed black line in each panel corresponds to the same set of parameters: L=100,γ=3,ρ=0.9.

We now take a broader look at the three recovery regimes, and the transitions between them. As suggested by Fig. 1, we expect dispersal d and local dynamics r to play similar but opposite roles in determining which type of recovery takes place. IR would occur for low d (high r), MR would occur for high d (low r), and RR may occur for intermediate values of both. We expect the system size L to have a similar role to r, since for smaller systems lower dispersal levels would be needed to homogenize the system. This general intuition is validated from the contribution of each regime to the recovery, as illustrated in Fig. 4a (see Appendix S3).

A final defining feature of the recovery regimes is the ratio between the timespans of local and regional recovery. Since the system may be far from its equilibrium locally, the timespan of local recovery may be very large, even infinite, depending on the disturbance intensity ρ and the system's nonlinearity, typified by γ in our model. By increasing either ρ or γ, local recovery in the disturbed region slows down considerably, while rescue dynamics are essentially unchanged. This is because rescue dynamics are driven by a flow of biomass from an undisturbed region, unaffected by the slow dynamics far from equilibrium. Therefore, as γ or ρ increases (Fig. 4b, c), RR (red region) grows at the expense of IR (green region), whereas MR (blue region) remains largely unchanged.

We can now formalize the heuristic reasoning described so far. Consider the dynamics of two adjacent uniform domains, one in equilibrium and the other disturbed. Due to dispersal, a smooth transition region (front) will form between these two domains. Such fronts have two main consequences for recovery: their motion into the disturbed domain leads to rescue dynamics, and hence RR, while if the fronts themselves are so large that they take over the entire system, then the system is in MR. If the fronts do neither of these things while recovery takes place, then the system is in IR. As detailed in Appendix S1, we use these implications of front dynamics (Zelnik and Meron 2018) on recovery, combined with a dimensional analysis (Legendre and Legendre 2012), to reach a prediction of the transitions between the recovery regimes. The result can be summarized by visualizing an axis of effective system size $\ell_{\mathrm{eff}}=L\sqrt{r/d}$. As formalized in Eq. (2), when the effective size decreases, the system transitions from IR to RR at $\ell_{\mathrm{eff}}=2u\tau_{0}$ and from RR to MR at $\ell_{\mathrm{eff}}=\lambda$

(2) $$\underset{\mathrm{IR}}{\underbrace{\ell_{\mathrm{eff}}>2u\tau_{0}}};\;\;\underset{\mathrm{RR}}{\underbrace{2u\tau_{0}>\ell_{\mathrm{eff}}>\lambda }};\;\;\underset{\mathrm{MR}}{\underbrace{\lambda >\ell_{\mathrm{eff}}}};\;\ell_{\mathrm{eff}}=L\sqrt{r/d}$$

The three physical parameters, L, r and d, are the same as described previously, while the three other quantities, u, λ, and $\tau_{0}$ are nondimensional properties of the model. The parameters u and λ are the nondimensional front speed and size, which do not depend strongly on model or disturbance properties (with typical values in the order of 1 and 10, respectively, see Appendix S4). On the other hand, $\tau_{0}$ is the recovery time without dispersal (scaled by r), with values that can change by orders of magnitude, depending on characteristics of both system (γ) and disturbance (ρ). To compute or estimate $\tau_{0}$, we set some arbitrary threshold to define full recovery (in our case N = 0.99 K), and measure the time needed (scaled by r) for the system to reach that threshold. We show in Appendices S2 and S4 some typical values and dependencies of u, λ, and $\tau_{0}$. It should be noted, however, that the definition of $\tau_{0}$ can be generalized beyond its specific definition of time to recovery from a single disturbance. More generally, it can be defined with respect to the fastest non‐spatial timescale that rescue dynamics must be compared to (and thus strongly depends on the dynamical scenario considered as will be evident in *Examples*). We will come back to this point in the discussion, and go into details in Appendix S2.

The prediction of regime transitions are shown in magenta lines in Fig. 4. The transitions themselves are not sharp: as a parameter is changed, a smooth transition occurs between the different regimes. The dimensional parameters d, r, and L all occur in the same term, implying a similar role in determining the recovery regime, as seen by the parallel lines in Fig. 4a due to changes in d and L. On the other hand, the changes in $\tau_{0}$ (Fig. 4b, c) due to increasing either γ or ρ lead to a larger region in parameter space where RR is dominant instead of IR. However, these same changes in γ and ρ do not have much of an effect on the values of u and λ, so that the RR‐MR transition remains largely unchanged.

## Examples

Our framework of recovery regimes can be applied to a wide range of questions in ecology, such as stability in a fragmented landscape and the interplay of synchrony and local extinctions. We detail here a few concrete examples and discuss their implications, to both demonstrate the application of the framework, and give credence to its generality. In doing this, we will also show that our initial assumptions, focusing on dynamics of a single species around a stable equilibrium in one spatial dimension, do not in fact limit the predictive power of the framework.

### Metapopulation stability

We start by an interpretation, in terms of our recovery regimes, of two experimental studies (Dai et al. 2013, Gilarranz et al. 2017) on the consequences of a localized population extinction on a metapopulation. The first (Dai et al. 2013) in order to consider a new spatial indicator of impending collapse, and the second (Gilarranz et al. 2017) to understand how modularity of the spatial network (connected patches) can buffer disturbances. Despite apparent similarities in the details of these studies, they are actually relevant in different recovery regimes, and hence in entirely different settings.

Dai et al. considered a bistable system, where the dynamics of populations close to the tipping point slow down, leading to larger “recovery length,” the size of the front connecting a disturbed region to a non‐disturbed region. Unlike the model used on *Conceptual Framework* and *Recovery Regimes*, the front dynamics here are those of a stable state invading another stable state. For such a system, however, the three recovery regimes still exist and the transitions between them are well predicted by our approach (see Appendix S4). In the study of Dai et al., a critical slowing down occurs when the system approaches the tipping point (Dakos et al. 2011), which effectively means that the system has smaller and smaller r. This slowing down thus leads the system from IR to RR and on to MR. In practice, however, the proposed indicator (increase in recovery length) is limited to RR since, in MR, this recovery length is larger than the system, so that it cannot be monitored.

On the other hand, Gilarranz et al. looked at how decimating a population locally leads to a propagation of the disturbance across the system. In this case, the assumption is that, due to spatial movement of individuals, the other parts of the system still suffer from the disturbance. As can be seen in the different recovery panels in Fig. 1, this behavior only occurs in MR, where the front is of a size that is comparable to the system. These two examples illustrate that knowledge of the recovery regimes can be paramount to estimate the applicability of ecological indicators (here front size and network modularity, respectively).

### Fragmentation scenarios

**Figure 5:**
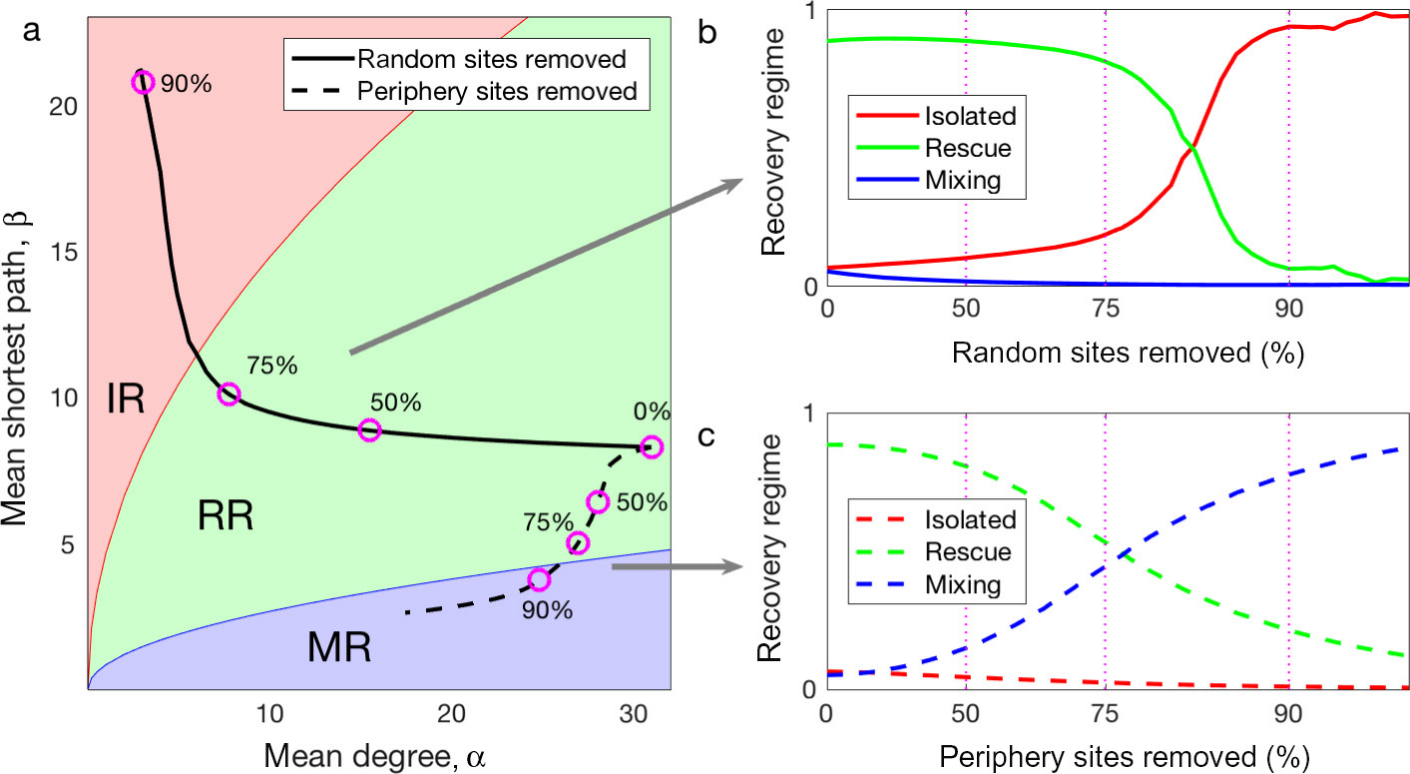
Fragmentation scenarios leading to a change in recovery regimes. (a) Parameter space of mean degree α (average number of links per site, as a proxy for dispersal coefficient d) vs. mean shortest path β (proxy for system size L). Overlaid are two fragmentation scenarios, one of randomly taking out sites (solid line) and one of taking out periphery sites (dashed line). Background colors are based on a prediction of transition lines between recovery regimes. (b, c) Contribution of the three recovery regimes to the overall recovery in a network, along the two fragmentation scenarios. Horizontal axis shows (in a logarithmic scale) the percentage of sites removed either (b) randomly or (c) from the periphery. Magenta circles in panel a show the removal of {0%,50%,75%,90%} of sites, with corresponding vertical lines in panels b and c. The initial network has 2,000 sites, with approximately 40 links per site on average, and r=1,d=2,γ=3,ρ=0.7,σ=0.5. See Appendix S3 for details.

A more complex spatial setting is that of habitat fragmentation, which is a leading factor of biodiversity loss (Saunders et al. 1991, Hanski and Ovaskainen 2000, Bennie et al. 2013). As both the number of habitat patches and the number of potential dispersal routes shrink due to fragmentation, the effects these changes have on various ecosystem properties, such as biodiversity and stability, are of high interest. Within our framework, we expect that such changes in spatial structure will change both the effective dispersal in the ecosystem, and its effective size. We can thus relate different scenarios of fragmentation to trajectories in a parameter space similar to that previously shown in Fig. 4a. In this case, the dispersal coefficient is related to the average link number per site α, and the system size to the average shortest path between two sites β (see Appendix S3 for details). As shown in Fig. 5a, by starting with a random spatial network, and removing sites from the system, different behaviors emerge. If sites are taken out from the periphery, then both α and β shrink (Fig. 5a, dashed line), leading to a tight‐knit system and therefore to MR (Fig. 5b). On the other hand, if the sites are taken out randomly, then β tends to grow (Fig. 5a, solid line), leading to IR (Fig. 5c). Thus, a change in spatial structure can lead to a complete switch in the system's dynamical behavior.

### Predator–prey synchronization

A different aspect of stability in a spatial setting, is that of synchrony and how it is affected by dispersal (Ripa 2000, Gouhier et al. 2010, Abbott 2011). Extinction due to synchrony of populations is of particular concern when the local dynamics do not have a stable equilibrium, as often seen in predator–prey interactions.

**Figure 6:**
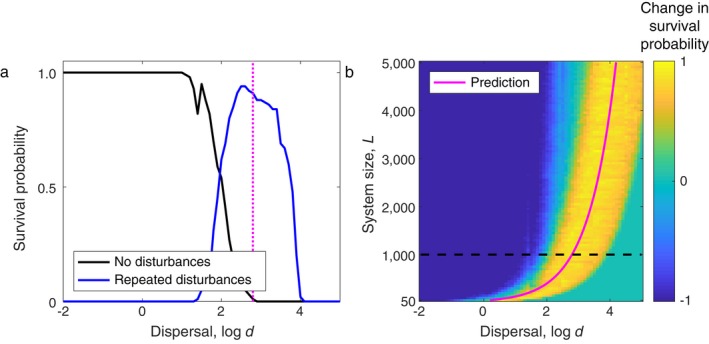
Localized extinction disturbances increase stability due to prevention of synchronization. Following Fox et al. (2017), we model populations of predator and prey in a spatial settings, which would go extinct without dispersal. (a) Survival probability of predator population as a function of dispersal coefficient d. Results for no disturbances (repeated disturbances) are shown in black (blue). (b) The change in survival probability (by the addition of repeated extinction disturbances) in a parameter space of dispersal coefficient d and system size L. Color gradient indicate the absolute effect of local extinctions on global survival probability (from dark blue, maximal reduction of survival probability to bright yellow, maximal increase in survival probability). The RR‐MR transition line (magenta) predicts where disturbances increase survival. Black dashed line shows the location of the cut shown in panel a, *L* = 1,000. See Appendix S3 for details.

This issue has been addressed recently by Fox et al. (2017) in an experimental setting, in which protist predator–prey metapopulations exhibit cyclic dynamics, which can, when dispersal is strong enough, lead to predator extinction (black line in Fig. 6a). Fox et al. found that inducing repeated localized extinctions of the predator can prevent its global extinction (blue line in Fig. 6a). This behavior occurs because these disturbances do not allow the system to become spatially synchronized, and thus vulnerable to global extinction. Such synchronization occurs faster with increasing dispersal, and when dispersal is strong enough for the system to be in MR, synchronization occurs almost instantaneously. Therefore, imposing localized disturbances in MR is harmful, as the disturbance frequency necessary to avoid synchronization would be so high as to directly lead to a global extinction. On the other hand, far from MR, synchronization is unlikely due to demographic stochasticity, so that imposing localized disturbances can, again, only be harmful. This leads to a hump‐shape relationship between survival probability and dispersal, as demonstrated by the blue line in Fig. 6a.

We therefore expect that localized disturbances will enhance survival probability in a specific setting, one where synchronization is weak enough so that localized extinctions can prevent it, and that rescue dynamics are strong enough to allow the system to recover from the disturbances themselves. Hence we predict that disturbances will increase survival probability around the RR‐MR transition, as can indeed be seen in Fig. 6 (see Appendix S3 for details). We are thus able to pinpoint the parameters for which this unintuitive phenomenon will occur. Moreover, this example highlights the fact that exotic behavior can be expected when a system is crossing the boundaries between recovery regimes.

### Metacommunity biomass productivity

**Figure 7:**
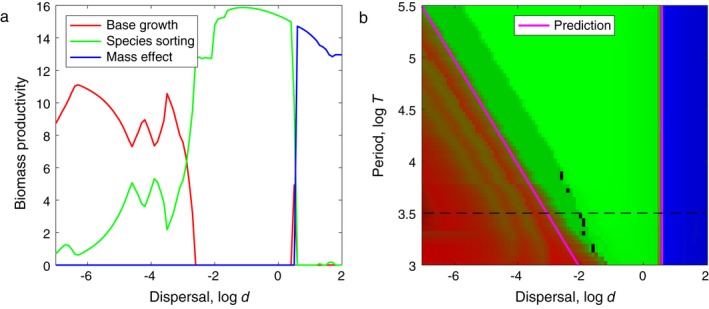
Biomass productivity in a metacommunity due to three different mechanisms. Following Thompson et al. (2017), we model an ecosystem of multiple species and a single resource in a spatial settings, with periodically changing conditions. (a) Biomass productivity as a function of dispersal coefficient d, where the (red, green, blue) lines correspond to three different mechanisms: base growth, species sorting, mass effect. (b) Parameter space of dispersal coefficient d and period of environmental change T, showing the different mechanisms. Magenta lines show the prediction of the transition lines between the three recovery regimes, which correspond well to the three mechanisms of biomass productivity. Black dashed line shows the location of the cut shown in panel a, *T* = 3,200. See Appendix S3 for details.

Our framework can be applied beyond the context of stability, for example by looking at productivity of metacommunities (Leibold et al. 2004). As shown in recent work by Thompson et al. (2017), along the dispersal axis and under periodically changing environmental conditions, there are three different mechanisms responsible for metacommunity biomass production: base growth, species sorting, and mass effect. For weak dispersal, biomass production is due to base growth, where communities are effectively isolated and the species composition does not follow changes in the environmental conditions. This behavior is contrasted with species sorting that takes place with higher dispersal, where species do not occur throughout space at a given time, but rather follow optimal conditions. Finally, for strong dispersal, species are abundant due to mass effects, by which species biomass from highly productive locations is dispersed across space. These mechanisms are illustrated in Fig. 7a, reproducing results of Thompson et al. using the same species traits and environmental properties, but a simpler spatial structure (see Appendix S3 for details). Although these three mechanisms of biomass production are not directly linked to stability, we propose that they are in fact equivalent to our three recovery regimes.

Base growth corresponds to IR, in which local dynamics (of competitive exclusion and recovery) are faster than spatial spread, so that each species occurs in certain regions and does not move from them. In contrast, species sorting corresponds to RR, where front propagation of populations is sufficiently fast to react to changing conditions. Finally, mass effect corresponds to MR, in which dispersal is significantly faster than local dynamics, so that biomass produced in favored regions spreads throughout the system.

To demonstrate this correspondence, we compare biomass production mechanisms with predictions of transitions between recovery regimes (as defined by Eq. (2)). As seen in Fig. 7b, the analytical transition lines (magenta) between recovery regimes predict the switch between biomass production mechanisms remarkably well. Note that for the calculation of the IR‐RR transition (Eq. (2)), the local timescale $\tau_{0}$ is defined by the period of environmental change (see Appendix S3 for details). This example highlights the fact that basic dynamical processes, such as those captured by our approach, underly the behavior of complex ecological systems. In our case, understanding these processes sheds light beyond recovery properties, predicting changes in ecosystem functioning.

## Discussion

By analyzing the response to a disturbance of a simple yet spatially explicit model, we could make powerful predictions about the stability properties of various complex spatial ecosystems, ranging from metacommunities in a changing environment to populations in a fragmented landscape. We highlighted three regimes of recovery, Isolated (IR), Rescue (RR), and Mixing (MR), and showed how these regimes depend on both the properties of the system, and of the disturbance that is imposed on it, thus mapping these regimes onto the space of system and disturbance parameters. The recovery processes involved in each of these regimes are qualitatively different. In MR, the system first homogenizes before local processes drive the system back to equilibrium, whereas in RR the recovery is driven by the propagation of biomass from undisturbed regions into the disturbed ones. The relationship between time to recovery and disturbance property (extent, intensity, and strength) thus differs substantially between regimes. More generally, any prediction about the effect of a given parameter on the system's stability will strongly depend on the regime the system is in.

To determine the three recovery regimes, there are two main constants to consider. The first constant is determined by three‐dimensional parameters of the system {d,r,L}, which combine to form a nondimensional constant $L\sqrt{r/d}$ that we call the effective system size. When this constant is very small, then the system is well mixed, and therefore in MR. When this is not the case, the limiting non‐spatial timescale $\tau_{0}$ (scaled by r), needs to be considered, as it determines the effective reach of rescue dynamics. When this effective reach is much smaller than the effective system size, then the system acts as multiple isolated sites, and hence is in IR. Otherwise, if the local timescales allow for a large effective reach (compared to effective size), then the system is in RR, in which undisturbed domains of the system are the main instigator of recovery and thus stability.

From a mapping of these regimes we could predict the effects of fragmentation and global change on basic features of ecosystem stability. What matters in this context is not only where the system is on the map, but where it is going. When global change moves the system closer to collapse, local dynamics slow down, and therefore the system is pushed towards MR. This is the premise of various early warning signals of catastrophic transitions (e.g., recovery length of Dai et al. 2013), made explicit within our framework. These indicators essentially measure how close the system is from MR (although being in this regime does not imply a collapse). Our mapping is especially useful when considering the combined effect of fragmentation and global change, since fragmentation may push the system towards IR, in contrast to the effect of slowing down due to global change. This means that the recovery regime may not change at all, so that early warning signals that measure the distance to MR will not detect the impending collapse. A much clearer picture is gained from knowledge of the system's trajectory on the map of recovery regimes. Following from our definition of recovery regimes, it follows that only in RR can there be an interplay of spatial scales, between the characteristic scale of the system and any scale that is imposed on it. This leads us to ask what phenomena are specific to this regime, especially since it is relatively less explored than IR and MR. In particular, the transition regions between RR and the other two recovery regimes, which may take place over a large range of parameter space, can be expected to be especially interesting and display exotic behavior, since here there is no clear timescale separation. Indeed, the surprising results shown by Fox et al. (2017), where local extinctions save the predator population from total extinction, well demonstrate this notion that the transition regions can show particular and unintuitive behavior.

In the presentation of our results we focused on the recovery of the system from a single disturbance. In this restrictive context, the definition of a non‐spatial timescale $\tau_{0}$ is a result of the interaction between local dynamics and disturbance intensity. More generally, the relevant definition of $\tau_{0}$ depends on the dynamical scenario in question, as shown in the example of biomass productivity (Fig. 7). In this case, the period over which conditions change defines the relevant non‐spatial timescale $\tau_{0}$. Other definitions of $\tau_{0}$ can be made, depending on the perturbations the system undergoes, as is detailed in Appendix S2. For instance, Yaari et al. (2012) investigated the spatial scaling of metapopulation persistence subject to demographic stochasticity, and universally found three distinct regimes across a gradient of dispersal. If one wanted to compare recovery regimes to the scaling regimes of Yaari et al., one would define $\tau_{0}$ as the local time to extinction, which must be compared to the time needed for rescue dynamics to take place.

This work demonstrates that despite inherent complexities in the dynamics of populations and ecosystems, strong qualitative predictions can be made from the analysis of a simple and generic model. Indeed, we studied the dynamics of a single species at equilibrium in a uniform one‐dimensional landscape, and considered its response to a single disturbance. However, our example of fragmentation scenarios shows that we can apply our methodology to more complex spatial structure. Moreover, the predictions shown for the predator–prey and the metacommunity systems clearly show that our framework can be applied to multiple species systems. These two examples also show that considering a system disturbed from equilibrium did not limit our predictive ability, as the predator–prey system was one where multiple disturbances occurred and the dynamics exhibited large oscillations, while the metacommunity system had no explicit disturbance at all, but rather a continuously changing environment. Overall these examples suggest that there are universal properties of ecosystem dynamics in a spatial settings, that can be unraveled from dimensional considerations.

Heterogeneities in space, time or species properties will impact the recovery regimes, however, if they are sufficiently strong. As noted in Appendix S5, both demographic and environmental noise (temporal heterogeneities) can have an effect on recovery regimes. In particular, they tend to make the transition between IR and RR more gradual, while having a minimal effect on the RR‐MR transition. Spatial heterogeneity can be expected to have a similar effect, having a strong impact on local dynamics when dispersal is weak, but less so when dispersal is strong, due to the spatial‐averaging effect of dispersal (Loreau et al. 2003). The case of species‐rich communities (heterogeneity of the community) is the most intriguing. In the most complex case of strong collective nonlinearities (e.g., complex succession dynamics, multiple equilibria, and so on) and heterogeneous dispersal abilities, more work is needed to say how to correctly apply and generalize our framework. Nonetheless, in addition to the specific examples given in *Examples*, there are at least two general cases for which our approach can be expected to directly apply. The first and more straightforward case is when a dominant species governs local dynamics (a simple case of strong heterogeneity). The second case, corresponding to moderate heterogeneity, would be a community comprised of many species with weak interactions and similar dispersal abilities. In this case, local dynamics will be a collective outcome of species interactions, but we may nonetheless define and predict transitions between recovery regimes with respect to a collective rate of local recovery.

Determining the relevant nondimensional constants that govern the recovery regimes is not always straightforward, but it sets a clear direction toward a deeper understanding of the spatial processes in a system of interest. For instance, our analysis of the fragmentation scenarios showed that the effective system size is essentially the average shortest path between two sites, rather than the total number of sites in the network. The issue of estimating the relevant parameters should be particularly interesting in the context of complex species interactions, due to collective emergent behavior. For instance, the characteristic timescale of a community, such as its local recovery time, may be an emergent property of the assembly process. Moreover, in the case of strong heterogeneities between species (e.g., substantially different dispersal abilities between trophic levels) it may be that more nondimensional constants are needed to faithfully describe the system's dynamics. For instance, a higher trophic level may spread much faster than its resource (McCann et al. 2005), leading to a combination of IR and MR.

Our study is essentially based on the presumption that we can learn a great deal on an ecological system by performing a simple calculation using our knowledge of its dimensional properties. The examples we have presented show that this claim has merit, and our methodology can indeed provide new insights into dynamics of spatial systems. This study takes us one step further towards a quantitative understanding of the response to disturbances of spatially extended ecological systems, drawing for the first time a clear link between the opposite cases of a well‐mixed system and a set of isolated sites. Considering the proliferation of theoretical and empirical studies, and the growing sets of observational and experimental data, being able to qualitatively compare different systems is of vital importance. Our study suggests a simple way of performing such a mapping, using limited information about a given system. Such an approach is relevant for both theoretical models and empirical data, paving the way towards a novel synthetic view on ecosystem dynamics in space.

## Data Availability

All simulations used to create the figures were made using Matlab with the library RDM: https://doi.org/10.5281/zenodo.1929096. Code that shows how simulations and calculations for each figure were made can be found at: https://doi.org/10.5281/zenodo.1928407.
