# Supplementary material for: The three regimes of spatial recovery
Source: Ecology. 2019 Feb 1;100(2):e02586. doi: 10.1002/ecy.2586 (PMC6375383; doi:10.1002/ecy.2586)
Supplement: Supplementary file 1 [file ECY-100-na-s001.pdf]

The three regimes of spatial recovery

Appendix S1: Dimensional analysis of front dynamics

Yuval R. Zelnik, Jean-François Arnoldi, Michel Loreau

Centre for Biodiversity Theory and Modelling, Theoretical and Experimental Ecology Station, CNRS and Paul Sabatier University, 09200 Moulis, France.

We describe here the analysis preformed on the model given by eq. 1, which will lead us to the predictions of recovery regimes. We use insight on the dynamics of fronts, combined with a dimensional analysis, as described below.

We begin by assuming that following a disturbance the system is composed of two domains, one at equilibrium, and a disturbed domain that is away and potentially very far from this equilibrium. Between these two domains smooth transitions regions (fronts) will form that will connect the two domains, due to dispersal. These fronts have two main consequences on recovery: their motion into the disturbed domain leads to rescue dynamics, and hence RR, while if the fronts themselves are so large they take over the entire system, then the system is in MR. If the fronts do neither of these things while recovery takes place, then the system is in IR. We can therefore predict that the transition between IR and RR occurs when the timespan of local recovery T_0 is the timespan of regional recovery T_R. The latter can be approximated by the ratio between the system size L and twice the front speed U (since two fronts take part in the recovery), leading to: $L/(2U) = T_R$. The transition between RR and MR occurs when the front size Λ is as large as the system, and hence $L = \Lambda$.

We now rewrite eq. 1 by considering one spatial dimension, and defining new non-dimensional variables for space, time and biomass $\{x,t,n\}$ instead of the old dimensional variables $\{X,T,N\}$, to produce eq. S1:

$$\partial_t n = nf(n) + \partial_x^2 n \tag{S1}$$

Where the definition of the new variables are: $x = X\sqrt{r/d}$; $t = Tr$; $n = N/K$, and we rewrite the F function as $f(n) = F(nK)$. Since eq. S1 has the same structure as eq. 1, they share the same properties, and in particular the same general dynamics of fronts. We therefore note the non-dimensional front speed and size as u and λ respectively. Due to dimensional constraints, we know that the relations between the new and old front properties are: $u = U/\sqrt{rd}$ and $\lambda = \Lambda\sqrt{r/d}$ (note that front properties do not depend on the system size L). Both u and λ are non-dimensional constants, and thus unrelated to any dimensional properties such as d, r or L. They therefore depend only on the type of local dynamics, as described by $f(n)$, and moreover, can be expected to be of the order of 1 if the local dynamics are not extremely nonlinear (see Appendix 4 for further exploration of this issue). We can also use this definition of u and λ, together with a non-dimensional constant of the recovery time without dispersal $\tau_0 = T_0 r$ to redefine the boundaries between the three recovery regimes:

$$L = \Lambda = \lambda\sqrt{d/r}$$

$$L = 2UT_0 = 2u\sqrt{dr}(\tau_0/r) = 2u\tau_0\sqrt{d/r}$$

We therefore have two inequalities for the transitions between the three regimes:

$$L\sqrt{r/d} < \lambda\,, \tag{S2a}$$

$$L\sqrt{r/d} > 2u\tau_0\,. \tag{S2b}$$

where the prediction is that if the first inequality holds then the system is in MR, if the second inequality holds then the system is in IR, and otherwise the system is in RR. Note that we implicitly assume that both inequalities cannot hold, namely that $2u\tau_0 < \lambda$.
