# Supplementary material for: The three regimes of spatial recovery
Source: Ecology. 2019 Feb 1;100(2):e02586. doi: 10.1002/ecy.2586 (PMC6375383; doi:10.1002/ecy.2586)
Supplement: Supplementary file 2 [file ECY-100-na-s002.pdf]

The three regimes of spatial recovery

Appendix S2: Derivation of non-spatial timescale τ_0

Yuval R. Zelnik, Jean-François Arnoldi, Michel Loreau

Centre for Biodiversity Theory and Modelling, Theoretical and Experimental Ecology Station, CNRS and Paul Sabatier University, 09200 Moulis, France.

1 τ_0 in different dynamical scenarios

τ_0 is the non-spatial timescale which competes with the rescue recovery dynamics. In the specific context of recovery from a single disturbance it arises when we ask which recovery is faster: the non-spatial one, or the one driven by rescue processes (front propagation). In a more general context the relevant question becomes whether the rescue dynamics have enough time to move through the disturbed region before any non-spatial process makes them irrelevant. Note that irrelevant here can be due to a positive change – where the disturbed region is recovered before the rescue dynamics played out – or due to a negative one, where the system is disturbed before rescue recovery could take place.

Consider a dynamical scenario of repeated localized extinctions, as in the predator-prey example (Fig. 6). To find τ_0 (which was not relevant to the mixing transition considered in Fig. 6), we should ask how long (on average) it takes before another such localized extinction occurs in the same place, such that any recovery that might have taken place will become irrelevant. If the relative size of local extinction events is σ, and the average frequency of disturbances is f, then we

can estimate $\tau_0 = r/(f\sigma)$, where we include r to get a non-dimensional value, relative to the characteristic, local dynamical timescale. A similar line of thought can be used for a different scenario of periodic environmental conditions (with period T) which are strong enough to determine growth and death, such as in the main text example of metacommunity biomass productivity (Fig. 7). In such a case τ_0 is even more straightforward to compute, it is simply $\tau_0 = r \cdot T$.

In some scenarios there could be competing non-spatial timescales. For example, if instead of localized extinctions, which set populations to zero locally, we have localized disturbances which set the density to a low but non-zero level. In that case there are two timescales: one related to the disturbance frequency, and the other to the recovery time from each disturbance. In general the faster timescale will be the relevant one, but if they are similar, then ignoring the other will likely become problematic.

When considering different dynamical scenarios, not only the derivation of τ_0 changes, but also the properties and interpretations of the three recovery regimes. This is evident in our example of metacommunity productivity, where the three regimes translate to different sources of biomass production. If we consider the case of repeated local extinctions (e.g. our predator-prey example), then the three regimes translate to qualitatively different behavior of population persistence. As an additional example, we hypothesize that the recovery regimes coincide with the persistence behaviors found in the study by (Yaari *et al.*, 2012), where increasing dispersal led to a logarithmic, exponential, then non-universal[1] scaling of persistence with system size.

2 Calculation of τ_0 for a single disturbance

To calculate τ_0 for the model used in the main text, we may focus on the non-spatial term, i.e. the ODE: $\frac{dn}{dt} = n^{\gamma+1}(1-n)$. We are looking for time to recovery. To do so we can separate the variables n and t and rewrite the ODE in an integral form $\int dt = \int \frac{n^{-\gamma-1}dn}{1-n}$. We then integrate it from $n(t=0) = 1-\rho$, the value of n immediately after the disturbance, until recovery at $n(\tau_0) = n_r$,

[1] i.e. dependent on specificities of local dynamics

with $n_r = 0.99$. This gives:

$$\tau_0 = \int_{1-\rho}^{n_r} \frac{n^{-\gamma-1}}{1-n} dn = \left[ln\left(\tfrac{n}{1-n}\right) + \sum_{m=1}^{\gamma} \tfrac{1}{m} n^{-m} \right]_{1-\rho}^{n_r} \tag{S1}$$

where the last equality holds for zero or positive integer γ. Thus, the exact solution for τ_0 is:

$$\tau_0 = ln\left(\frac{1-\rho}{\rho}\right) + ln\left(\frac{n_r}{1-n_r}\right) + \sum_{m=1}^{\gamma} \frac{1}{m} \left[n_r^{-m} - (1-\rho)^{-m} \right] \tag{S2}$$

An approximate expression, with a simpler form can be made by dropping some terms, giving:

$$\tau_0 \approx ln\left(\frac{1-\rho}{\rho}\right) - ln(1-n_r) + \frac{1}{\gamma}(1-\rho)^{-\gamma} \tag{S3}$$

which shows, in a slightly more transparent way, the dependence in γ (non-linearities) and ρ (disturbance intensity). These expressions, as well as numerical results, are plotted against ρ for several values of γ in Fig. S1.

References

Yaari, G., Ben-Zion, Y., Shnerb, N.M., and Vasseur, D.A. (2012). Consistent scaling of persistence time in metapopulations. *Ecology*, 93, 1214–1227.

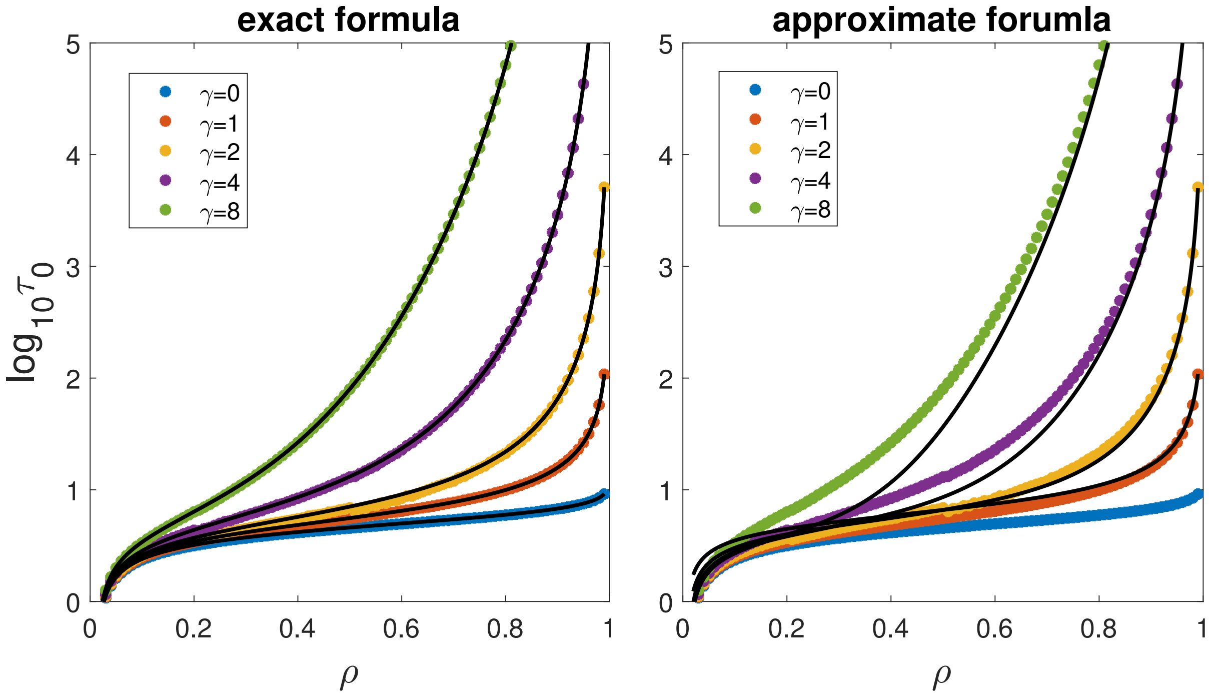

Figure S1: **Time for recovery without dispersal τ_0 for different values of ρ and γ.** Left panel compares numerical results to the analytical expression in eq. S2, showing an exact match. Right panel shows an approximation that holds well for large values of ρ, given by eq. S3.
