# Supplementary material for: The three regimes of spatial recovery
Source: Ecology. 2019 Feb 1;100(2):e02586. doi: 10.1002/ecy.2586 (PMC6375383; doi:10.1002/ecy.2586)
Supplement: Supplementary file 3 [file ECY-100-na-s003.pdf]

The three regimes of spatial recovery

Appendix S3: Details on simulations and calculations made to produce the figures in the main text

Yuval R. Zelnik, Jean-François Arnoldi, Michel Loreau

Centre for Biodiversity Theory and Modelling, Theoretical and Experimental Ecology Station, CNRS and Paul Sabatier University, 09200 Moulis, France.

This appendix gives the full information on the calculations and simulations used to produce the figures in the main text. Section 1 described the model of a one-dimensional metapopulation model (used for figures 1-4), while sections 2, 3 and 4 give details for figures 5, 6 and 7 respectively.

1 Analysis of uniform metapopulation model

The results shown in Fig. 1-4 are all based on eq. S1, in one spatial dimension.

$$\partial_T N = rN(1-N/K)(N/K)^{\gamma} + d\nabla^2 N \tag{S1}$$

In general, there are seven parameters relevant for these results. The five parameters of the system are: local dynamics rate r, dispersal coefficient d, carrying capacity K, nonlinearity γ, and system size L. Two parameters define the disturbance: its local intensity ρ, and its spatial extent σ, where the overall strength of the disturbance is $s = \sigma\rho$.

For Fig. 1, in the three simulations shown the parameters r and d were varied to showcase the three different recovery regimes. (b) IR was shown using $r = 4, d = 0.01$, (c) RR was shown using $r = 1, d = 1$, and (d) MR was shown using $r = 0.01, d = 8$. Other parameters were held constant: $K = 1, \gamma = 3, L = 80, \rho = 0.85, \sigma = 0.5$.

In Fig. 2 two simulations were used, where (b,c) the localized disturbance had $\sigma = 0.4, \rho = 1$, while (d,e) the global disturbance had $\sigma = 1, \rho = 0.4$, so that the overall strength of the disturbance was the same with $s = 0.4$. Other parameters were held constant: $r = 1, d = 1, K = 1, \gamma = 3, L = 400$.

For Fig. 3 the three parameter spaces shown were calculated with different levels of dispersal: (a) $d = 10^{-3}$ (b) $d = 10^{1}$ (c) $d = 10^{5}$. In all, the extent σ and intensity ρ were varied between 0 and 1. Other parameters were held constant: $r = 1, K = 1, \gamma = 3, L = 500$.

For Fig. 4, in all three parameter spaces shown the dispersal coefficient d was varied (in log scale) between 10^{-6} and 10^{-6}. For panel (a) the system size L was varied (in log scale) between 10 and 10000, while $\gamma = 3, \rho = 0.9$. For panel (b) the nonlinearity parameter γ was varied between 0 and 6, while $L = 100, \rho = 0.9$. For panel (c) the disturbance intensity ρ was varied between 0.01 and 1, while $L = 100, \gamma = 3$.

For both Fig. 1 and Fig. 4 the transition lines of IR-RR and RR-MR are shown, based on the analytical formula $L\sqrt{r/d} = \lambda$ and $L\sqrt{r/d} = 2u\tau_0$, respectively. To do this three non-dimensional constants were estimated numerically, using simulations of the non-dimensional version of of equation S1 (by setting: $r = 1, d = 1, K = 1$). By simulating a front between $n = 1$ and $n = 0$, the front speed u was estimated as $u = 0.37$ and the front size was estimated as $\lambda = 11$. Note that here we always used $\gamma = 3$ and $\rho = 1$ (in effect, since one domain had $n = 0$), since the dependence of both λ and u on these changes in minimal. On the other hand, for estimation of τ_0 both γ and ρ were very significant. Here we looked at a comparable system, but we set the dispersal to zero $d = 0$, and estimated the recovery time (to just below $n = 1$, specifically to $n = 0.99$) when the entire system is set to a value $1 - \rho$. For $\gamma = 3, \rho = 0.85$ (as used in Fig. 1) we had $\tau_0 = 132$, while for $\gamma = 3, \rho = 0.9$ (as used in Fig. 4a) we had $\tau_0 = 400$.

2 Fragmentation scenarios

For the analysis of fragmentation scenarios, we used the same general modeling scheme of eq. S1, as detailed in the previous section, but instead of a one-dimensional system, a spatial network was used. In this network setup, we used link-wise dispersal, so that each link allows for a proportional amount of biomass to disperse, and hence the more links a site has, the more biomass will disperse between it and its neighbors. Each link is identical, and so is each site in its local conditions. Dispersal level was defined so that if a network is made of a one dimensional chain, and the number of sites in the network M is the same as the size of a one dimensional system L, then the dispersal (and in general all other results) will be identical between these two systems (assuming dispersal is not too strong). This was done simply for an easier comparison with results of a one dimensional system, and has no bearings on the results since it amounts to a scaling of the dispersal coefficient. In practice, the dynamical equation per site is:

$$\frac{d}{dt}N_i = rN_i(1-N_i/K)(N_i/K)^\gamma + d\sum_j^M \delta_{ij}N_j - dN_i\sum_j^M \delta_{ij} \tag{S2}$$

where N_i is the biomass at site i, and δ_{ij} is the connectivity matrix, with values of 1 if there is a link between sites i and j, and 0 otherwise.

The networks used were constructed in the following way. M sites were given random locations (uniform distribution) in a two-dimensional space within a circle. For each site, the c closest neighboring sites were chosen, and if a link did not already exist, then it was formed between the initial and neighboring site with a probability p. In all simulations the parameters used were $M = 2000, c = 40, p = 0.5$, so that the average number of links per site was approximately 31.

To simulate different fragmentation scenarios, two methods of removing sites were used. The scenario where periphery sites were removed (solid lines in Fig. 5) was done by looking at the distance of each site from the center of the whole network. Here the locations are defined as the ones originally used to construct the network. The sites that were farthest from the center were removed, in a non-random fashion. The second scenario was that of removing random sites

(dashed lines in Fig. 5). Here an iterative scheme was used to avoid a disconnection of the network to several clusters due to the site removal. In this way the network remains one cluster so that biomass can propagate from each site to any other site. This was done so that the effective size of the network could be assessed faithfully using the measure of the average shortest path (see below).

The iterative scheme is as follows. At each iteration step, m sites are chosen at random. If their removal will not disconnect the network, then the removal is enacted. If it will disconnect the network, then no sites are removed in this step, and instead the value of m is halved, as long as it is not already smaller than a threshold value of z. The iterations are repeated until the required number of sites are removed, or up to a maximum number of iterations i. If this maximum is reached (not enough sites were removed) then the remaining number of sites to be removed are chosen at random, ignoring the issue of network disconnection. For a network site of $M = 2000$ sites, we started with m that is the total number of sites to be removed, with a threshold value z of 0.002 of the overall number of sites to be removed, and maximum iteration number $i = 500$. Using this iterative scheme, the network does not disconnect until over 90% of sites are removed, whereas a completely random removal would disconnect the network at around $70\% - 80\%$ of sites removed. At the same time, other properties of the network, such as its recovery dynamics, do not change considerably.

The two axes shown in Fig. 5a are the average link number in the x-axis and the average shortest path in the y-axis. The average link number is a proxy for the dispersal coefficient of the system d. It is calculated as the number of links a given site has, averaged over all sites in the network. The average shortest path is a proxy for the size of the system L. It is measured by calculating the minimal number of sites that are needed to connect between two given sites, averaged over all possible pairs of two sites. If a network is not connected (i.e. some sites are not reachable by other sites), then the average shortest path is defined as infinity. In Fig. 5a such networks are not shown.

The simulations of the fragmentation scenarios were preformed similarly to those of the one-dimensional system, except that eq. S2 was used, with δ_{ij} which are the result of a network creation

and site-removal processes described above. The disturbance was imposed by choosing an initial site at random, and going through its neighbors and their neighbors and so forth, until M_σ sites were chosen, such that $M_\sigma = \sigma M$. We used $\sigma = 0.5$, so that $M_\sigma = 1000$ sites were affected by a disturbance. Other parameters used were $r = 1, d = 2, K = 1, \gamma = 3, \rho = 0.7$. The results shown were averaged over 100 different networks that were created and underwent the process of site-removal, with a different randomization key. For each network, 10 recovery processes were measured, each with a different initial site for the disturbance.

The transitions between different regimes, as shown in Fig. 5a, are based on the prediction detailed in the previous section. This result has to be augmented to fit the settings of network fragmentation. First, a relation needs to be drawn between the effective dispersal coefficient d_e, which is used instead of d, and the average number of links $\hat{n}$. Since the number of links in a chain topology (which is equivalent to a 1D setting given in the previous section) is 2 links per site, we need to normalize by this value, so that $d_e = \frac{1}{2}\hat{n} \cdot d$. Second, for the effective system size L_e, we use the average shortest path $\hat{p}$ as a proxy for system size L. Since the networks we use are set in a two-dimensional (2D) circular space, and the average distance between two points in a such a 2D space is approximately a quarter of the diameter of such a region, we need to normalize by this value. Hence we use: $L_e = 4\hat{p}$. Finally, the homogenizing effect of the front (relevant for the RR-MR transition) needs to be normalized since we are dealing with a 2D space (as opposed to the 1D setting in the previous section). The proper normalization for the front size (λ_e) is not trivial, and we leave it for future work. However, using geometric considerations we can hypothesize that the normalization constant will be π, and this value indeed gives a good agreement with a mapping of recovery regimes on multiple networks with different parameters that we preformed (not shown). We therefore use $\lambda_e = \pi\lambda$. Putting all these together we have our prediction for the two transition lines: $L_e\sqrt{r/d_e} = \lambda_e$ and $L_e\sqrt{r/d_e} = 2u\tau_0$

3 Predator-Prey system

The analysis of the effect of local disturbances on the stability of a predator-prey metacommunity was done by following previous work on such a system that used both experiments and simulations (Fox *et al.*, 2017). We use the third model described in this study, a version of the Rosenzweig-MacArthur predator-prey model. The model used is:

$$\partial_t A = r(1 - A/K)A - \omega AB/(\phi + A) + d\nabla^2 A, \tag{S3a}$$

$$\partial_t B = \varepsilon AB/(\phi + A) - \gamma A + d\nabla^2 B. \tag{S3b}$$

where A is the prey biomass and B is the predator biomass, and the parameters values used are (following the original paper): $r = 1.85, K = 35920, \omega = 25.5, \phi = 11364, \varepsilon = 12.4, \gamma = 2.07$ and d is varied (see below). The model was simulated in one spatial dimension of length L with periodic boundary conditions.

To allow extinctions we check the biomass density at each location every time-step, and if it is smaller than a threshold value of $N_{th} = 1$ then its value is set to 0 (biomass densities typically go over 10^3, so that this has a minimal effect when populations levels are high). In each simulation, the initial population of both predator and prey was randomly chosen from a uniform distribution between 0 and 10^4 without correlations in space. The simulation was run for a constant length of time $T = 100$, and the predator population was deemed to have survived if it is non-zero anywhere in the system. The simulations were repeated 100 times with different randomization keys, and the results of survival probability derived from averaging over these simulation results. Two simulation conditions were compared, one where no local disturbances were induced, and the other where 100 disturbances occurred throughout the simulation time in constant intervals, a disturbance every 1 unit of time. This comparison was done while changing two system parameters: d was varied (in log scale) between 10^{-2} and 10^5 and L was changed between 50 and 5000.

The prediction shown in Fig 6 of the RR-MR transition uses an estimation of the front size. This was done by taking a system without any predator or prey population, and seeding a small domain

of the system with both predator and prey (with density $A = B = 1$). While the dynamics in the original domain tend to be chaotic, both prey and predator slowly invade the bare-region. Thus a front is formed after a sufficient time which has the same properties regardless of initial conditions, and its size can be calculated. Since the front in this system is non monotonic and includes spatial oscillations, its estimation is not very accurate, but its size can still be approximated, and a value of 40 was used to produce the transition line shown in Fig. 6 (magenta curve).

4 Biomass productivity in a metacommunity

The analysis of biomass productivity in a metacommunity was done by following previous work on such a system (Thompson *et al.*, 2017). The model used is based on older work of the spatial insurance hypothesis (Loreau *et al.*, 2003), and uses a system with M species N_i that differ in their use of a single resource R. The model equations are:

$$\partial_t N_i = N_i(ec_i R - m) + d\nabla^2 N_i \;, \quad i = 1..M \tag{S4a}$$

$$\partial_t R = I - lR - R\sum_i^M (c_i N_i) + d\nabla^2 R. \tag{S4b}$$

where $e = 0.2, m = 0.2, I = 150, l = 10, M = 9, L = 100$, dispersal coefficient d is varied between simulations (in log scale) in the range 10^{-7} and 10^{-2}, and the consumption rates c_i are varied during the simulation itself to simulate temporal and spatial changes in the environmental conditions. This change in environmental conditions occurs with a period of T which is varied (in log scale) between 10^3 and $10^{5.5}$. Each species has a different periodic curve of its consumption as a function of time and space, so that by either moving through the whole system (distance L) or waiting a time period of T, the species will experience all possible conditions specified by this curve. More explicitly:

$$c_i(x,t) = \frac{1.5 - |H_i - E(x,t)|}{10} \;\; ; \;\; H_i = \frac{i}{M}$$

$$E(x,t) = \frac{1}{2}\left[sin(2\pi(\frac{x}{L} + \frac{t}{T})) + 1 \right]$$

Note that the environmental conditions change with a speed of $v = L/T$

For each point in the parameter space of d and T (Fig. 7), one simulation was made. The initial conditions used were that of uniform densities of all species and resource with an arbitrary value of 1. The simulation was run for a time of $10T$ to minimize the effect of transients, and then an additional time of $5T$ was run, over which the biomass productivity was analyzed (see below). In order to allow species to go extinct, throughout the simulation, at every times-step the biomass of each species is checked at every location, and if it lower than a threshold value of $N_{th} = 0.1$ its value is set to 0.

Biomass productivity was defined as the change in biomass at a given time (that is, the right hand side of eq. S4), except for the contribution of mortality (ignoring the term $-mN_i$), integrated over some region (either the entire system, or some part of it). The biomass productivity was divided between three sources: base-growth, species-sorting and mass-effect. If a species had non-zero densities at every location of space throughout the entire simulation (after the transient run-time of $10T$ was finished), then all its biomass growth was considered to be due to mass-effect. If a species in a certain location had gone extinct ($N_i = 0$) at any time point in the simulation (but was non-zero in some other time) then all the biomass of this species that was produced throughout the simulation was considered to be due to species-sorting. Productivity in any other case (namely, if a species is always present at a given location, but is not present throughout the entire system) was considered to be due to base-growth.

The prediction shown in Fig 7 of the IR-RR and RR-MR transitions (magenta curves) uses three dimensionless constants: front size, front speed and recovery time. In the context of this system, there is no explicit disturbance, but rather the constant change of environmental conditions effectively acts as a disturbance. Therefore, instead of a recovery time we use as a proxy the period over which the environmental conditions change, so that $\tau_0 = T$ (see also Appendix S2). To estimate the front properties we consider the same system but with a constant environment ($c = 0.1$), put in one species in a small domain, and follow its propagation into the bare region. From this simulation we can estimate the front properties as: $\lambda = 40$ and $u = 0.54$.

References

Fox, J.W., Vasseur, D., Cotroneo, M., Guan, L., and Simon, F. (2017). Population extinctions can increase metapopulation persistence. *Nat. Ecol. Evol.*, 1, 1271–1278.

Loreau, M., Mouquet, N., and Gonzalez, A. (2003). Biodiversity as spatial insurance in heterogeneous landscapes. *Proc. Natl. Acad. Sci.*, 100, 12765–12770.

Thompson, P.L., Rayfield, B., and Gonzalez, A. (2017). Loss of habitat and connectivity erodes species diversity, ecosystem functioning, and stability in metacommunity networks. *Ecography*, 40, 98–108.
