# Supplementary material for: The three regimes of spatial recovery
Source: Ecology. 2019 Feb 1;100(2):e02586. doi: 10.1002/ecy.2586 (PMC6375383; doi:10.1002/ecy.2586)
Supplement: Supplementary file 4 [file ECY-100-na-s004.pdf]

The three regimes of spatial recovery
Appendix S4: Relation between local dynamics and front properties

Yuval R. Zelnik, Jean-François Arnoldi, Michel Loreau

Centre for Biodiversity Theory and Modelling, Theoretical and Experimental Ecology Station, CNRS and Paul Sabatier University, 09200 Moulis, France.

The results and methodology presented in the main text hold in a wide range of systems. In an effort to make more explicit what generalizations can be made, and what are some of the limitations, we focus here on some technical aspects of the local dynamics considered and on their ramifications to the behavior of invasion fronts.

1 Models

We use the following four models throughout the text below. The first model is the same one used in the main text, in a non-dimensional form:

$$\partial_t n = n(1-n)n^{\gamma} + \partial_x^2 n \tag{S1}$$

The second model is an example where local dynamics show alternative stable states. The local growth has an Allee effect, so that without dispersal, densities below a threshold $n < \alpha$ decay to

the stable bare state $n = 0$.

$$\partial_t n = n(1-n)(n-\alpha) + \partial_x^2 n \quad ; 0 < \alpha < 1/2 \tag{S2}$$

The third model shows dynamics which are slower near the stable equilibrium $n = 1$ than near the unstable one $n = 0$.

$$\partial_t n = n(1-n^q) + \partial_x^2 n \quad ; 0 < q < 1 \tag{S3}$$

The fourth model is similar to the first one, except that we artificially add, near the bare state, a small linear term of approximately $C \cdot n$. This term is used to allow a linear analysis around the unstable equilibrium $n = 0$, which is problematic in the first model when $\gamma > 0$. This last model reads

$$\partial_t n = n(1-n)(n^\gamma + C) + \partial_x^2 n \tag{S4}$$

For all models, a dimensional form is recovered by multiplying local dynamics by a rate r, the diffusion term by a dispersal coefficient d, and consider, for the system, a physical length of L.

2 Pulled and pushed fronts

A major part of this study revolves around understanding how dynamics of fronts play a role in the response of a system to perturbations. Our study focuses on general dimensional considerations and the interplay between local and spatial processes, without emphasis in the main text on front properties beyond their immediate dimensional consequences (i.e. size and speed). However, for some of the discussion below, it is useful to introduce a distinction between two types of fronts: pulled and pushed (van Saarloos, 2003). Pulled fronts move due to the leading edge of the front "pulling" the bulk behind it. In contrast, pushed fronts move due to the dynamics inside the front structure, "pushing" the leading edge into the invaded state, which in the models considered is the bare state $n = 0$.

If the front is pulled, then the leading edge – which has a low density – controls the front

speed, which can be calculated from a linear analysis around the unstable state. A known example is that of logistic growth with diffusion in a one-dimensional space (eq. S1 with $\gamma = 0$). A classical calculation (van Saarloos, 2003) reveals the speed to be $u = 2$, which stems from the linear term in the logistic model. On the other hand, if a front is pushed, its speed is controlled by the whole behavior of the front, which is inherently nonlinear (as it connects between two equilibria), and therefore a calculation of front speed itself also requires a nonlinear analysis. For instance for the Allee effect model of eq. S2, the speed of the front is: $u = \sqrt{2}(1/2 - \alpha)$ (Lewis & Kareiva, 1993). At $\alpha = 1/2$ the front is stationary (Maxwell point). For larger values of α it switches directions, so that the bare state invades the populated state.

Our study focuses on systems where the dynamics around the undisturbed equilibrium are faster (or as fast) as the dynamics in the disturbed region. In this case we expect fronts to be pushed since the growth at the leading edge is slow by construction, and hence a faster recovery is achieved when the whole front region spills out into the invaded domain. By contrast, if the local dynamics in both the disturbed and undisturbed domains are of comparable speed, then the situation is more ambiguous, and the system can have pulled fronts. In the context of the model we consider in the main text, logistic growth ($\gamma = 0$) shows pulled fronts with a speed of $u = 2$. However, as we show in Fig. S1, for $\gamma > 0$ this is not the case, and the system has pushed fronts.

3 Front properties

Different front properties can be relevant to the dynamics of the system, and therefore also to its recovery regime. We focused on two such properties, namely the size and speed of the front, due to their direct connection with our dimensional considerations. For completeness, we consider here an additional front property: the time t needed for the front to form.

In Fig. S2 we show numerical estimates for these three front properties, for the three models given by eqs. S1-S2-S3. The front speed u remains constant and equal to 2 for the third model, while for the two other models it slows down so that $0 < u < 2$. We see that λ and t behave

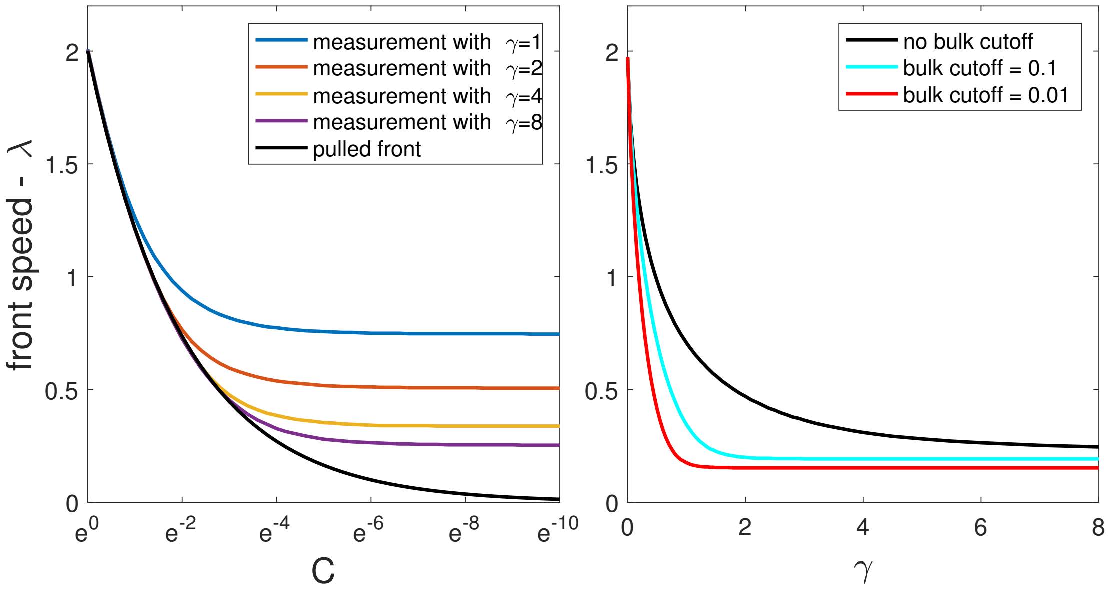

Figure S1: **Demonstration that the model used in the main text has pushed fronts for $\gamma > 0$.** Left panel uses the model in eq. S4 (which adds a linear term Cn), and compares the numerical results of front speed for different values of γ (colors) compared to the the analytical prediction (black) of a pulled front (which does not depend on γ). One can clearly see that for all values except $C = 1$ the actual front speed is faster than the pulled one, implying a pushed front. In particular, as C is smaller, the model becomes closer to the original definition, and clearly the pulled speed goes to zero while the actual speed remains constant. Right panel shows numerical calculation of front speeds for the original model (eq. S1), where in black is a normal simulation, and for cyan and red a cutoff is enacted on the system, so that the density of N cannot be higher than a given value (0.1 and 0.01, respectively). For all values except $\gamma = 0$ (where all three lines meet), the effect of the cutoff is to slow the front speed, indicating that the bulk of the front is pushing the front along, and hence that it is a pushed front.

similarly: they both remain constant for the bistable model, while changing considerably for the first and third models. In those cases the nonlinearity, and in particular the ratio between the speed of dynamics around the stable equilibrium and far from it, become more extreme as γ is increased or q is decreased. Both λ and t show a clear trend of growing with this increasing ratio. Note however, the extreme range of values considered here for γ (q), going from 1 to 100 (1/100). For instance to increase λ by a factor 10, we must increase γ also by factor 10. On the other hand, the range of d over which the system is in the rescue regime will increase by a factor of 10^{10}. It is thus evident that although there are clear trends in the behavior of front properties due to increasing

nonlinearity, they are relatively weak, and are not expected to have a substantial effect in most systems.

4 Ramifications to regime transitions

As noted in section 2, the model studied in the main text (eq. S1) shows a broad range of behaviors, from comparable speeds around the stable equilibrium and far from it for $\gamma = 0$, to dynamics where the recovery rate without dispersal of a disturbed state is effectively zero, thus mimicking bistable dynamics. In the main text we showed how this translates to different recovery regimes. Here we extend this range of behavior even further on both ends, by comparing the results for eq. S1 with those of eq. S2 (bistable dynamics) on the one hand, and to eq. S3 (slower dynamics around equilibrium) on the other.

In Fig. S3 we look at the contribution of MR, and compare the effects of slowing down the dynamics far from the stable equilibrium (increasing γ in eq. S1), to slowing down the dynamics around it (decreasing q in eq. S3). As evident from Fig. S3 , in both cases increasing the nonlinearity increases the MR domain. This increase is slow, as we previously noted, so that if we increased γ from 0 to 10, we would see the IR-RR transition move by approximately 10 orders of magnitude, while the RR-MR transition will move by less than 1 order of magnitude. When comparing the accuracy of our prediction for RR-MR transition, however, a clear distinction between the two models can be made. While the prediction works well for the first model, even for very strong non-linearities, it is increasingly inacurate as non-linearities grow in the model given by eq. S3. In other words, while the change in the transition follows the growth of λ in eq. S1, it is not the case for eq. S3. Before explaining why, let us first note that, as γ increases, the increasing time to front formation studied in the previous section does not seem to have any visible effects on the mixing transition between regimes.

Let us now comment further on the discrepancy between prediction and simulations of the mixing transition as q decreases in eq. S3, i.e. as near-equilibrium dynamics become increasingly

slow in comparison to the low-density growth rate. In this case the front formation and propagation is not the limiting recovery behavior. The situation is in fact much simpler than in other models: it is near the populated equilibrium that dynamics are the slowest and limit recovery. Thus, a linear analysis near the populated state ought to be sufficient to predict the mixing transition. In a linear model of the form $\partial_t w = -\beta w + d\partial_x^2 w$, one can decompose any spatial profile into Fourier modes and the mixing transition is attained when non-trivial Fourier modes (spatial heterogeneities) are absorbed at a rate comparable to the absorption of the uniform state. In the simplest case where the landscape is a ring of length L, the Fourier modes are $\varphi_k(x) = e^{i\frac{2\pi k}{L}}$; $k = 0, 1, 2, ...$

If we decompose any spatial profile w as $\sum_k w_k(t) \cdot \varphi_k(x)$, the amplitude $w_k(t)$ of Fourier modes will be exponentially damped following $\partial_t w_k = -(\beta + d(\frac{2\pi k}{L})^2) w_k$. The slowest non-trivial mode is the first and predicts a mixing transition when $\beta = d(\frac{2\pi}{L})^2$. In our third model, in its dimensional form, a linearization around the populated state gives $\beta = qr$, so that we can predict a transition when:

$$d/r = q(\frac{L}{2\pi})^2 \tag{S5}$$

As we can see in Fig. S3, this prediction is accurate.

We can also compare the dynamics of our model (eq. S1) at high γ (very slow dynamics far from equilibrium) with those of a truly a bistable model (eq. S2). As can be seen in Fig. S4, the general behavior for these two models is similar, with all three regimes occurring throughout the parameter space of d and ρ, with the MR for high d, while with lower d the system is in either RR for high ρ or IR for low ρ.

References

Lewis, M. and Kareiva, P. (1993). Allee Dynamics and the Spread of Invading Organisms. *Theor. Popul. Biol.*, 43, 141–158.

van Saarloos, W. (2003). Front propagation into unstable states. *Phys. Rep.*, 386, 29–222.

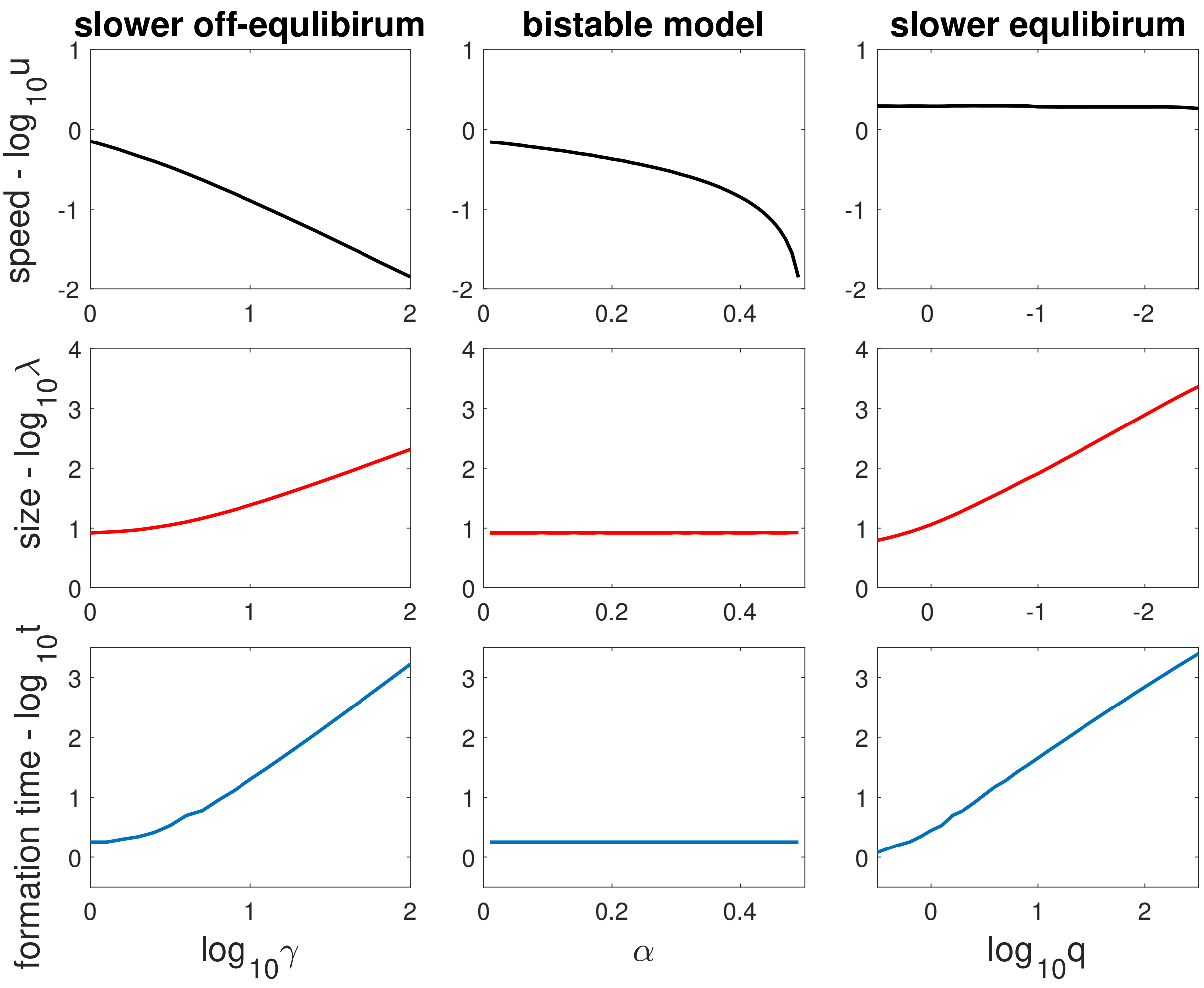

Figure S2: **Properties of fronts for different local dynamics.** Top row shows the front speed, middle row shows the front size, and bottom row shows the time it takes for the front to form. Left column shows these for the model presented in the main text with slower dynamics far from equilibrium (eq. S1), middle column for a bistable model (eq. S2), and right column for a model where the dynamics around the equilibrium are slower (eq. S3). Front speed is estimated by first waiting long enough for the front to form, then following the shrinking size of the bare domain for some time, and dividing the change in domain size by the time period. Front size is estimated by measuring the size of a the domain that has values of n between the two equilibria within some threshold (arbitrarily chosen as 0.05). Front formation time is estimated by following the front development, comparing between fronts at different times by aligning them so that the value of $n = 0.5$ is at the same location, and calculating the square of the difference between the two fronts. The formation time is defined as the time for 90% of the change to take place, starting from initial conditions of two uniform and opposing domains.

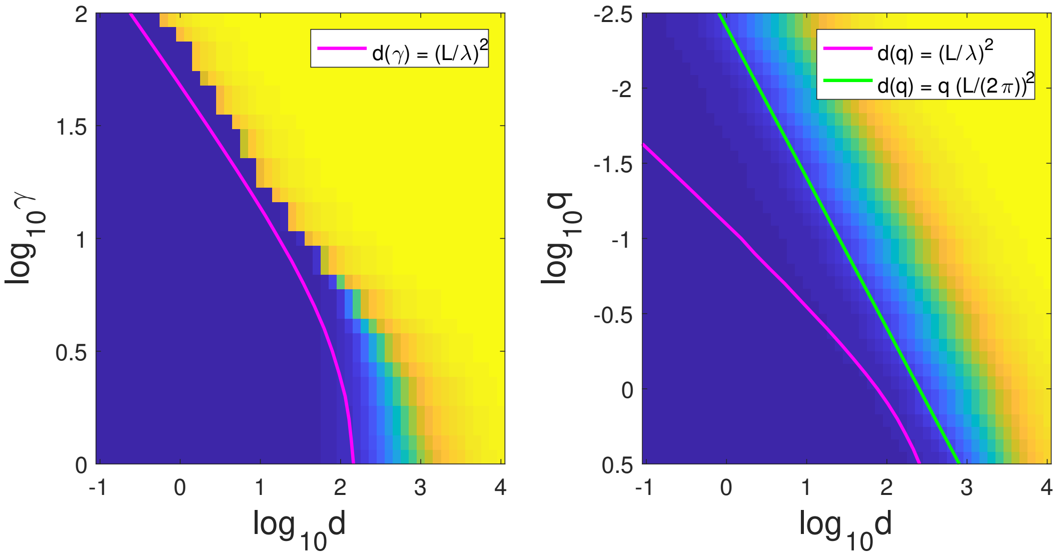

Figure S3: **The mixing transition for stronger nonlinearities** Parameter spaces of dispersal (d) in x-axis and nonlinearity (γ in eq. S1 and q in eq. S3, on left and right panels respectively) in y-axis, showing the contribution of the Mixing Regime to recovery. Magenta lines show prediction using the front size λ, while green line shows the appropriate prediction of eq. S5 for a system where local dynamics are slower around the equilibrium. Parameter values used are: $\rho = 1, \sigma = 0.5, r = 1, L = 100$.

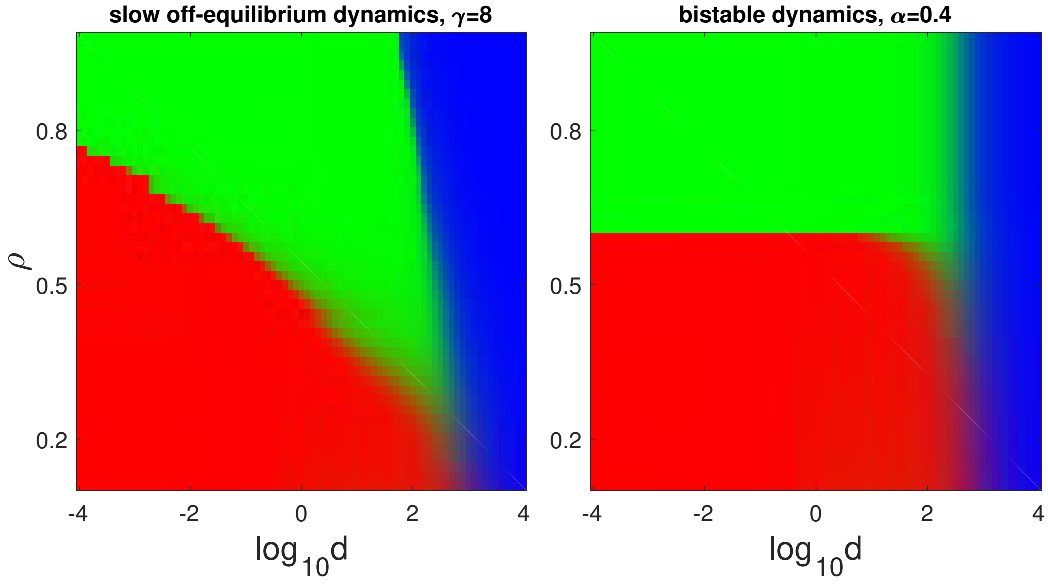

Figure S4: **Comparison between very slow local dynamics far from equilibrium (eq. S1) and bistablity of local dynamics (eq. S2).** The three regimes are shown, as a function of dispersal d and disturbance intensity ρ, for two the models showing different local dynamics. Parameter values used are: $\gamma = 8, \alpha = 0.4, \sigma = 0.5, r = 1, L = 100$.
