# Supplementary material for: The three regimes of spatial recovery
Source: Ecology. 2019 Feb 1;100(2):e02586. doi: 10.1002/ecy.2586 (PMC6375383; doi:10.1002/ecy.2586)
Supplement: Supplementary file 5 [file ECY-100-na-s005.pdf]

The three regimes of spatial recovery

Appendix S5: Effects of noise

Yuval R. Zelnik, Jean-François Arnoldi, Michel Loreau

Centre for Biodiversity Theory and Modelling, Theoretical and Experimental Ecology Station, CNRS and Paul Sabatier University, 09200 Moulis, France.

The effects of stochasticity, in particular demographic noise, is of special interest in ecology. We test its effect on two representative examples from our modeling framework. We add noise using stochastic differential equations (SDE), and compare the behavior, in terms of recovery regimes, of these systems to those of their deterministic counterpart. We first consider the case of $\gamma = 2$ (slow low-density growth, leading to a pushed front – see Appendix S4), and on the other hand, the case $\gamma = 0$, i.e. logistic growth (fast low-density growth, leading to a pulled front – see Appendix S4).

In Fig. S1 we compare simulations along the dispersal axis with and without demographic noise. One immediate observation is that for strong dispersal the discrepancy between cases disappears. This occurs because, for strong dispersal, the effective size of the population increases (as farther populations become more connected). We see, however, that the transition zones between the three regimes do not show a clear change due to the addition of noise, but that the transitions become more gradual.

We can also compare the results for demographic noise with those for environmental noise which scales proportionally with the number of individuals. We choose the strength of the environmental noise to be comparable, near the populated state, to that used for the study of demographic

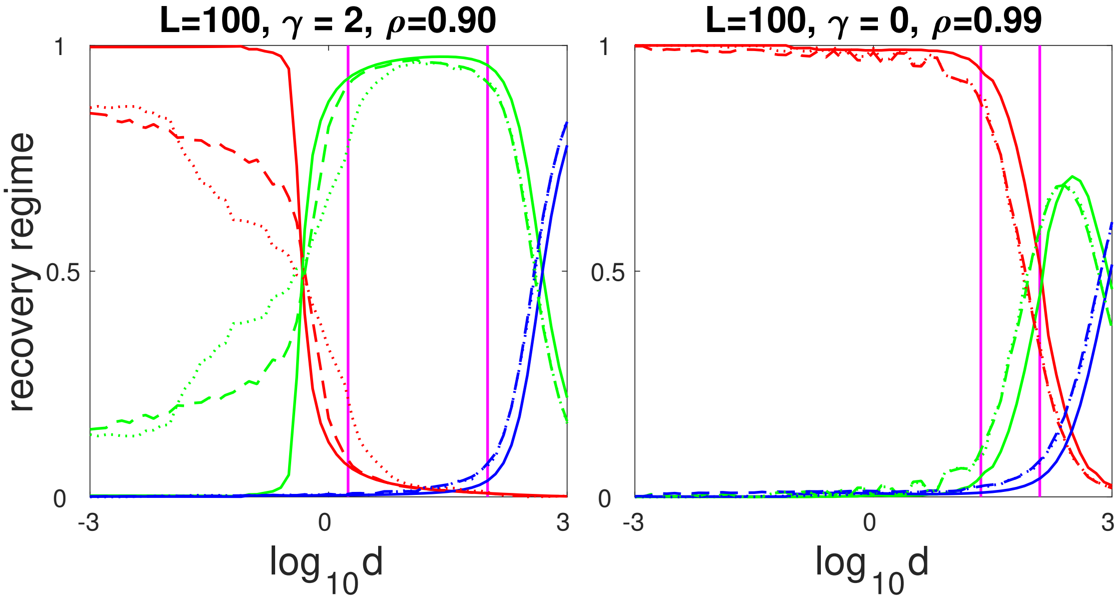

Figure S1: **The effect of demographic noise on the contribution of the three recovery regimes as a function of dispersal.** Horizontal axis shows (in a logarithmic scale) the dispersal coefficient, while the vertical axis shows the relative contribution of each recovery regime (red for IR, green for RR, blue for MR). Solid lines are for simulations without noise, while the dashed and dotted lines show the results for populations with an equilibrium density of 10000 and 1000 respectively. Vertical magenta lines show predictions based on simulations without noise. Results with demographic noise were averaged over 20 simulations per value of d. The simulations were made with an Euler-Maruyama scheme where at each time step δt a noise term was added to the integration: $w\xi\sqrt{\delta t}\sqrt{n}$, where n is the biomass density, ξ is a normally distributed random variable, and w equals 10^{-2} ($10^{-3/2}$) for the dashed (dotted) lines. Parameter values: $\sigma = 0.5, r = 1, L = 100$ for both panels, with $\gamma = 2, \rho = 0.90$ ($\gamma = 0, \rho = 0.99$) for left (right) panel.

noise. As seen in Fig. S2, the same trends discussed above apply here as well: noise has a stronger effect on regimes for weaker dispersal and its main effect is a more gradual transition between regimes, without any apparent change in where the regimes themselves occur.

It is interesting to note that the fact that both types of noise have a similar effect implies that the main effect of noise is not through its influence on front properties (since demographic noise can slow down the front speed considerably, while this need not be the case for environmental noise). In fact, the addition of noise in both scenarios had a minimal effect on both front speed and front size, when compared to its effect on the non-spatial timescale τ_0. This strong effect on τ_0 is

essentially due to the fact that with the addition of noise an Allee effect emerges as low populations can go extinct (leading to infinitely long τ_0). This is also consistent with other observations of how nonlinearity of local dynamics affects τ_0 in a much stronger fashion than it affects the front properties (see Appendix S4).

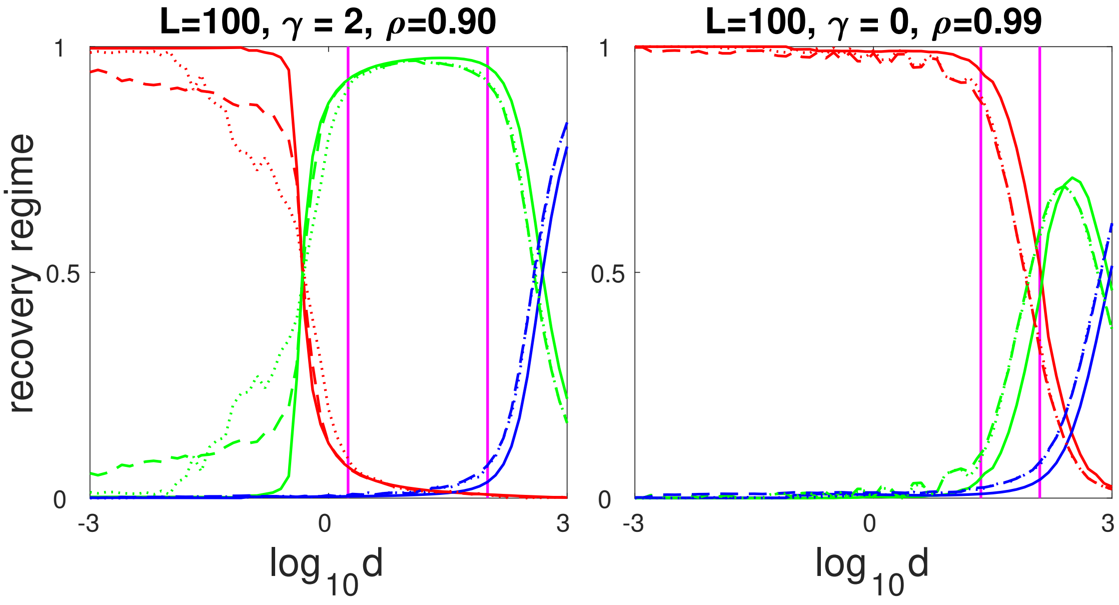

Figure S2: **The effect of environmental noise on the contribution of the three recovery regimes as a function of dispersal.** Horizontal axis shows (in a logarithmic scale) the dispersal coefficient, while the vertical axis shows the relative contribution of each recovery regime (red for IR, green for RR, blue for MR). Solid lines are for simulations without noise, while the dashed and dotted lines show the results for noise with a strength that is linearly proportional to the current density, with a coefficient of 10^{-2} and $10^{-3/2}$ respectively. Vertical magenta lines show predictions based on simulations without noise. Results with environmental noise were averaged over 20 simulations per value of d. The simulations were made with an Euler-Maruyama scheme where at each time step δt a noise term was added to the integration: $w\xi\sqrt{\delta t}\cdot n$, where n is the biomass density, ξ is a normally distributed random variable, and w equals 10^{-2} ($10^{-3/2}$) for the dashed (dotted) lines. Parameter values: $\sigma = 0.5, r = 1, L = 100$ for both panels, with $\gamma = 2, \rho = 0.90$ ($\gamma = 0, \rho = 0.99$) for left (right) panel.
